# Spontaneous Hypoglycemia in a Non-diabetic Patient: A Diagnostic and Therapeutic Conundrum

**DOI:** 10.7759/cureus.65467

**Published:** 2024-07-26

**Authors:** Karnika Mahendiran, Swathy Moorthy, Lakshmi M, Emmanuel Bhaskar

**Affiliations:** 1 Internal Medicine, Sri Ramachandra Medical College and Research Institute, Sri Ramachandra Institute of Higher Education and Research, Chennai, IND

**Keywords:** islet cell hypertrophy, nesidioblastosis, insulinoma mimic, spontaneous hypoglycemia, diabetes

## Abstract

Hypoglycaemia is a medical emergency requiring an immediate intervention to prevent neuroglycopenic symptoms such as confusion, seizures, and coma. While evaluating for the cause of hypoglycemia, after excluding common causes like insulin use or sepsis, other causes involving endogenous hypoglycemia need to be evaluated. A cause to be considered is nesidioblastosis. This rare entity is also known as non-insulinoma pancreatogenous hypoglycemia syndrome. There have been instances where this disorder has been mistaken as insulinoma due to the characteristics shared by the two. Here, we present a case of a non-diabetic male experiencing symptoms of giddiness and palpitations for the past two years who had been extensively evaluated to rule out insulinoma and was diagnosed with nesidioblastosis.

## Introduction

Adult-onset hypoglycemia can be due to various causes, and a thorough evaluation is warranted. Certain differential diagnoses to be considered include oral hypoglycemic agents, insulin, consumption of alcohol, critical illness like renal, cardiac, hepatic failure, or sepsis, pituitary insufficiency or adrenal insufficiency, or endogenous surplus of insulin or insulin-like growth factors [1]. Endogenous hyperinsulinemic hypoglycemia is a diagnosis of exclusion. Such a diagnosis in adulthood is called noninsulinoma pancreatogenous hypoglycemia syndrome, while the condition presenting in childhood is called persistent hyperinsulinemic hypoglycemia of infancy [1].

## Case presentation

A 44-year-old gentleman with no known comorbidities presented to our hospital with complaints of giddiness and palpitations for a prolonged duration of two years that had not been evaluated. The episodes of giddiness were accompanied by excessive sweating and palpitations. However, his palpitations were not accompanied by chest pain, breathlessness, or syncope, and no inciting event for the palpitations was noted. The last episode of giddiness with palpitations occurred two days before the presentation.

The patient admitted having an increased appetite compared to his baseline and noticed increased daytime sleepiness. The patient had no bowel or bladder disturbances during the episodes. The patient had a history of smoking and alcohol consumption. The patient had no history of surgeries in the past. Both of his parents had diabetes and hypertension. On examination, vitals were stable, with a BMI of 32 kg/m^2^. The patient had a darkening of skin around his neck representing acanthosis nigricans. Cardiac, respiratory, abdominal, and neurological examinations were within normal limits. Laboratory investigation revealed a fasting blood glucose of 35 mg/dl and glycated hemoglobin (HbA1C) of 5.1 (Table 1). The complete blood count and renal and liver parameters were normal.

**Table 1:** Laboratory investigations ACTH: adrenocorticotropic hormone; FT4: free T4; TSH: thyroid stimulating hormone

Laboratory investigation	Value	Reference Range
C-peptide	16.69	1.1-4.4 ng/ml
6 am serum cortisol	13.35	4.8-19.5 mcg/dl
Fasting insulin	158.3	2.6-24.9 mIU/l
ACTH stimulation test- serum cortisol in one hour	25.21	2.47-11.9 mcg/dl
Serum prolactin	20.39	4.04-15.2 ng/ml
FT4	1.07	0.93-1.7 ng/dl
TSH	2.340	0.27-4.2 mIU/L
Serum calcium	10.1	8.8-10.6 mg/dl

**Figure 1:**
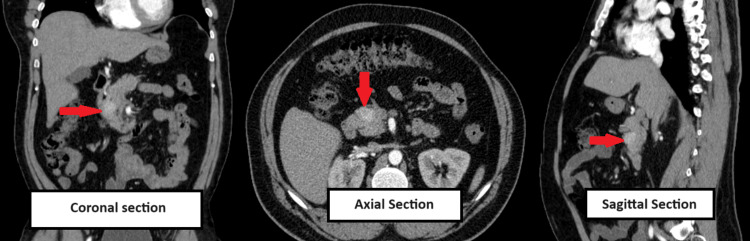
CECT showing small ill-defined lesion with mild arterial enhancement in the head of pancreas raising suspicion of neuroendocrine tumour of pancreas. Red arrow indicates lesion in pancreas. CECT: contrast-enhanced computed tomography

Ultrasound of the abdomen was normal. Contrast-enhanced computed tomography (CECT) of the abdomen revealed a small, ill-defined lesion of 1.6x2.1x2.3 cm with mild arterial enhancement in the head of the pancreas, suggesting a neuroendocrine tumour of the pancreas (Figure 1).

**Figure 2:**
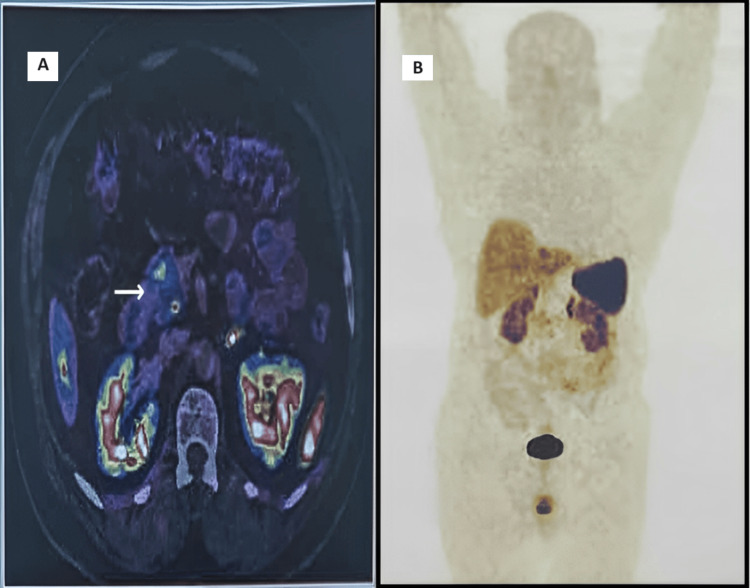
(A) Ga DOTANOC PET showing well-defined enhancing lesion in the head of the pancreas; (B) Image showing no evidence of abnormal Ga DOTANAC avid accumulation elsewhere in the body PET: positron emission tomography; Ga: gallium

Further biochemical workup revealed an elevated C-peptide level and elevated fasting insulin. Serum cortisol on the adrenocorticotropic hormone (ACTH) stimulation test was elevated, and elevated serum prolactin was noted. Gallium (Ga)-DOTANOC positron emission tomography (PET), was done revealing a well-defined enhancing lesion in the head of the pancreas with low-grade Ga-68 DOTANOC activity, suggestive of poor somatostatin receptor-expressing lesion, likely a neuroendocrine tumor, with no evidence of Ga-68 DOTANOC avid disease elsewhere in the body (Figure 2).

**Figure 3:**
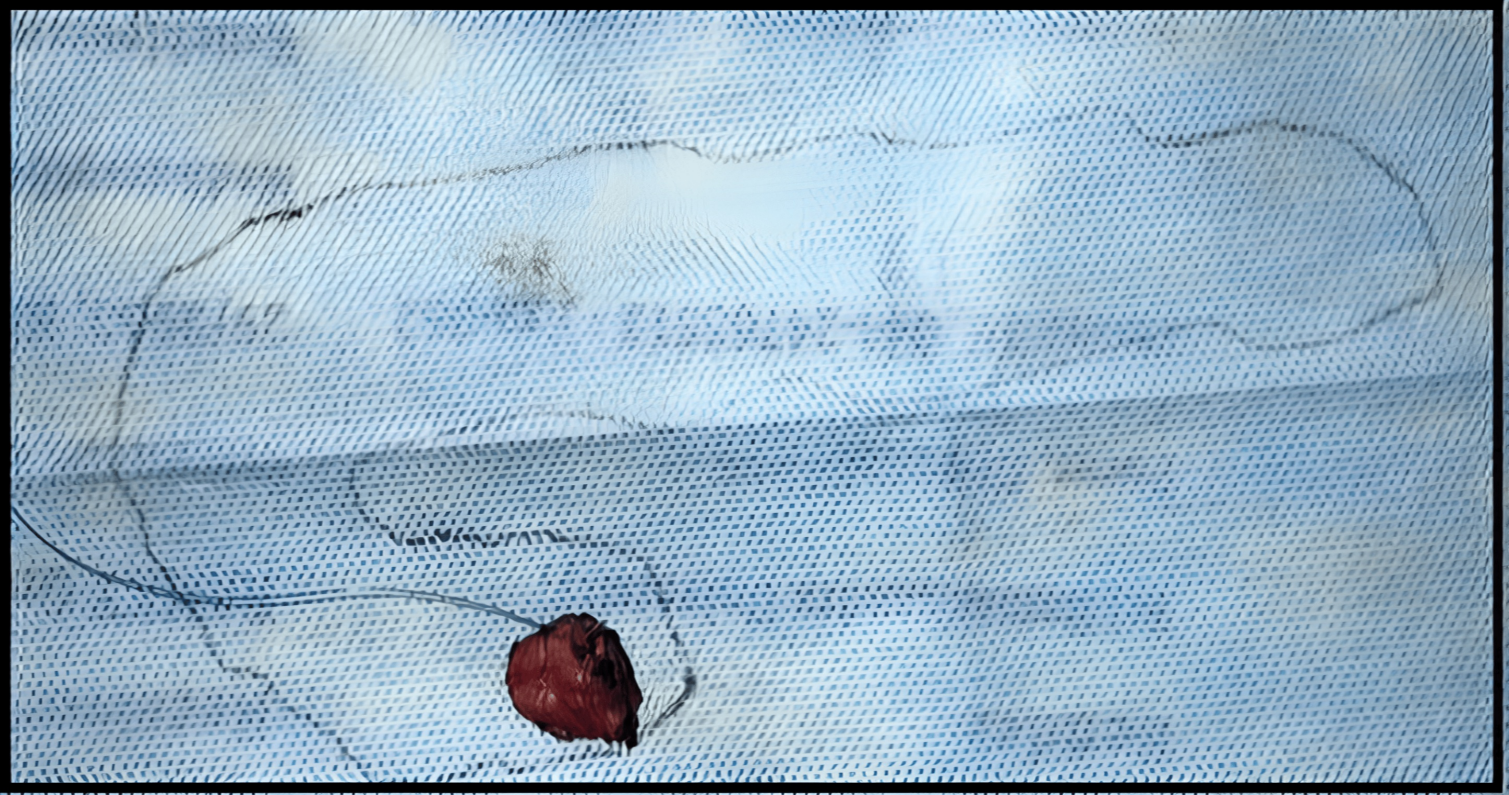
Postoperative photograph of enucleated neuroendocrine tumour on a background sketch of the pancreas, showing the relative location and size of the tumour

Throughout the hospital stay, the patient had multiple episodes of hypoglycemia, which were managed by dextrose boluses and maintenance dextrose infusions. The patient underwent enucleation of the pancreatic neuroendocrine tumour (Figure 3).

**Figure 4:**
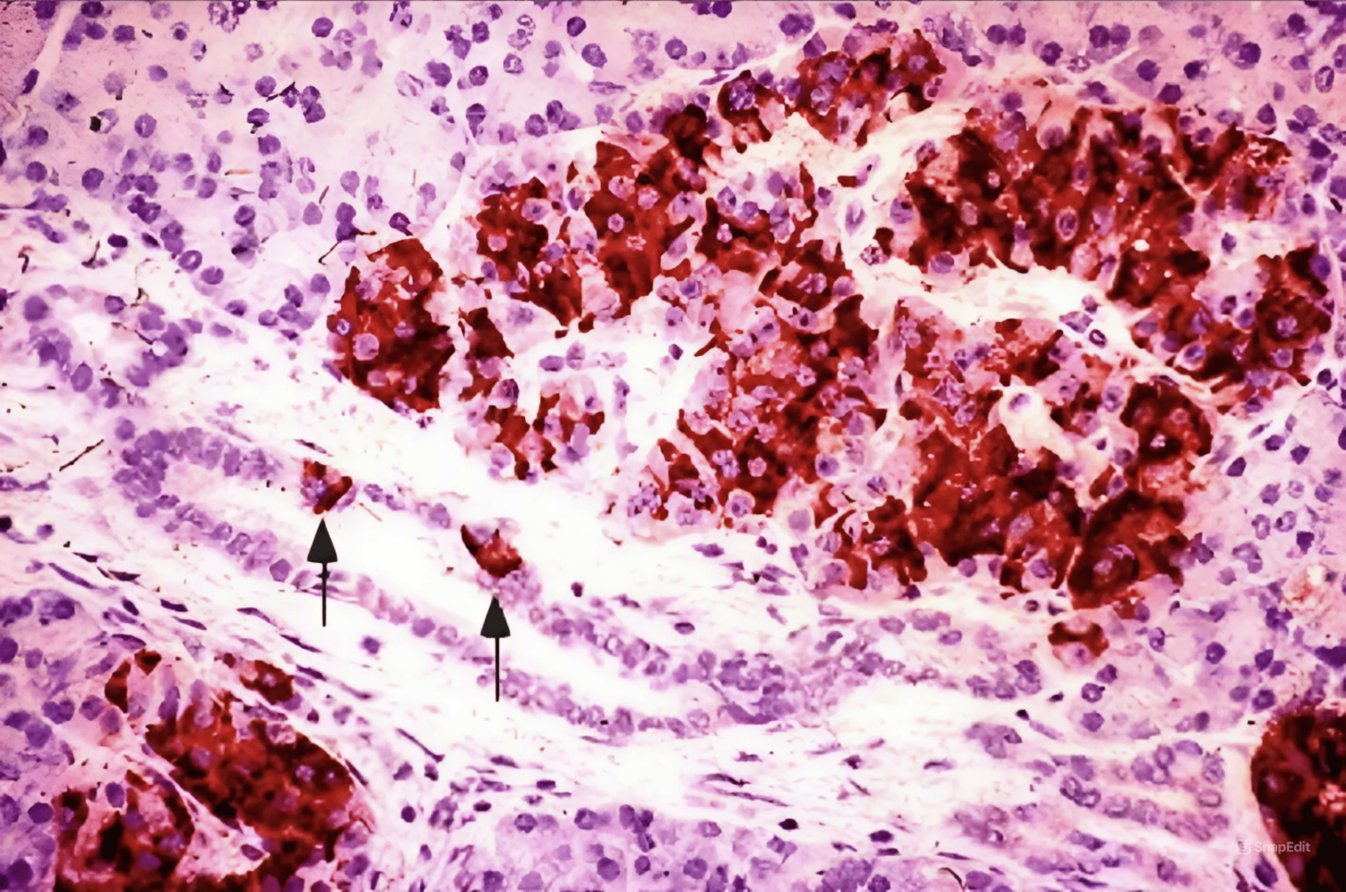
Positive insulin immunostaining of a hypertrophied islet (upper right) and two beta cells budding from an acinar duct (arrows)

Intraoperative findings revealed no evidence of an obvious exophytic or morphologically distinct tumour from the rest of the pancreas. Histopathology of the tissue revealed features suggestive of islet cell hyperplasia (nesidioblastosis) (Figure 4).

The patient continued to have persistent hypoglycemic episodes in the postoperative period, which warranted dextrose administration. Hence, there was a need for resection of the head of the pancreas. The patient was discharged with a drain in situ. At discharge, the patient received tab diazoxide three times daily. The patient needed readmission owing to persistent abdominal pain with vomiting. Repeat CECT abdomen revealed an intrabdominal peripancreatic collection; hence, computed tomography-guided pigtail catheterization with pus drainage was done and the patient was discharged. The patient regularly monitored blood sugar levels at home, and further hypoglycaemic episodes were managed by diet alone. He was planned for a total pancreatectomy in case of clinical worsening; however, the patient did not require the same due to diligent monitoring. Quality of life improved with reduced episodes of hypoglycemia, and the patient is currently on management with diazoxide.

## Discussion

Hypoglycemia is a life-threatening condition that can present with features of sweating, palpitations, fatigue, seizures, and coma. In the evaluation of healthy subjects, differentiating endogenous hyperinsulinemia or hyperinsulinemic hypoglycemia from functional or reactive hypoglycemia is required. The most commonly suspected cause of endogenous hyperinsulinemic hypoglycemia is insulinoma [2]. Endogenous hyperinsulinemia or hyperinsulinemic hypoglycemia is defined by low blood sugar levels with concurrently high insulin and C-peptide levels [3].

Insulinomas are the most common cause of hyperinsulinemic hypoglycemia, accounting for roughly 90% of the cases [4]. Other causes like functional beta cell disorders, Hirata’s disease, or insulin secretagogues have also been observed. Hirata’s disease is due to antibodies against insulin receptors or against insulin itself. The widely accepted hypothesis is the double-phase mechanism, which proposes that the autoantibodies bind to insulin or its receptor, preventing their interaction and producing hyperglycemia. However, the antibodies soon dissociate, resulting in a proper receptor-substrate interaction, thus lowering blood sugar levels irrespective of the existing blood glucose concentration [5]. Functional beta cell disorders are a group of less well-defined entities characterized by endogenous insulin secretion in excess quantities whose presentation varies widely among patients. They have been frequently associated with bariatric surgeries like Roux-en-Y bypass [6]. These disorders are frequently included under nesidioblastosis, which describes the neoformation of nesidioblasts. Nesidioblastosis is a histopathological term whose diagnosis can only be established by biopsy. Clinical features are described as non-insulinoma pancreatogenous hypoglycemia syndrome (NIPHS). The adult form is less common than the form occurring during infancy. 

The pathogenesis of nesidioblastosis is not well established. Certain mechanisms have been proposed, including excess growth factor production or their receptor expression [7], beta cell function dysregulation [8], or certain genetic variants. In those undergoing bariatric surgeries, it has been attributed to increased beta-cell trophic factors [6]. Environmental triggers have been proposed as causes for late-onset NIPHS [9]. Islet cell hypertrophy is reported frequently [9]. Certain authors have observed vascular ectasia within the islets among patients with hyperinsulinemic hypoglycemia [7]. Electrophysiological studies conducted on nesidioblastosis-derived cells showed that depolarization and action potentials were registered on the cell membrane even at low glucose concentrations [10].

Adult-onset nesidioblastosis is similar to insulinoma clinically and biochemically. The need for differentiating between the two arises as therapeutic strategies are vastly different. Conventional radiological imaging fails to diagnose occult insulinoma and nesidioblastosis; hence, newer diagnostic tools like glucagon-like peptide 1 receptors, Ga-68 DOTANAC PET, and selective arterial calcium stimulation (SACS) have emerged [11]. SACS can aid in determining the extent of surgical intervention. SACS was not done in our case, as the result from the Ga-68 DOTANAC PET/CT made the diagnosis of nesidioblastosis very evident. There is a current need for highly sensitive and specific functional imaging procedures, as SACS is invasive.

Treatment for this condition is based on the severity of the disease. In cases with mild symptoms, nutritional modification with proper spacing of carbohydrate meals with a low glycemic index may be attempted. The alpha-glucosidase inhibitor acarbose is useful in cases that are refractory to a low-carbohydrate diet. It slows down carbohydrate resorption. In some cases, glucocorticoids and beta-blockers were used as they induce hyperglycemia [12]. Diazoxide, which inhibits insulin secretion by activating ATP-dependent potassium channels, may be useful. In cases with more severe symptoms, a graded pancreatectomy may be required. A consensus has not been reached on the extent of resection required. Surgical intervention is associated with a set of postoperative morbidities. Our patient went through a similar diagnostic and therapeutic approach and is currently being managed with diazoxide with no further hypoglycemic episodes due to diligent meal planning. Apprehension may almost always mount up from the patient's perspective when they develop hypoglycemia after pancreatic lesion surgery, which is the case with our patient as well. However, with proper counseling and meticulous team efforts from the general physician, endocrinologist, surgical gastroenterologist, and dietician, along with the efforts of the patients and their families, patients can be managed without pancreatectomy.

## Conclusions

Diagnosis of nesidioblastosis presenting as recurrent spontaneous hypoglycaemic episodes has proven difficult due to its complex clinical presentation and onset across various age groups. Further research is required as conservative management is limited and surgical treatment options lead to significant morbidity and mortality in patients. Less invasive therapeutic options are required in the future, which could help conserve pancreatic functions.
